# Non-Deterministic Modelling of Food-Web Dynamics

**DOI:** 10.1371/journal.pone.0108243

**Published:** 2014-10-09

**Authors:** Benjamin Planque, Ulf Lindstrøm, Sam Subbey

**Affiliations:** 1 Institute of Marine Research, Tromsø, Norway; 2 Institute of Marine Research, Bergen, Norway; Phillip Island Nature Parks, Australia

## Abstract

A novel approach to model food-web dynamics, based on a combination of chance (randomness) and necessity (system constraints), was presented by Mullon et al. in 2009. Based on simulations for the Benguela ecosystem, they concluded that *observed patterns of ecosystem variability may simply result from basic structural constraints within which the ecosystem functions*. To date, and despite the importance of these conclusions, this work has received little attention. The objective of the present paper is to replicate this original model and evaluate the conclusions that were derived from its simulations. For this purpose, we revisit the equations and input parameters that form the structure of the original model and implement a comparable simulation model. We restate the model principles and provide a detailed account of the model structure, equations, and parameters. Our model can reproduce several ecosystem dynamic patterns: pseudo-cycles, variation and volatility, diet, stock-recruitment relationships, and correlations between species biomass series. The original conclusions are supported to a large extent by the current replication of the model. Model parameterisation and computational aspects remain difficult and these need to be investigated further. Hopefully, the present contribution will make this approach available to a larger research community and will promote the use of non-deterministic-network-dynamics models as ‘null models of food-webs’ as originally advocated.

## Introduction

Natural living systems are characterised by a high level of complexity, which results from the diversity of biological components at many levels of organisation (molecules, cells, organs, individuals, species, communities) and from the diversity of possible interaction types (physical, chemical, trophic, behavioural, cognitive). In addition, many biological interactions are non-linear, include feedback loops, and biological systems display a remarkable ability to constantly adapt and reconfigure themselves. Such systems, which display high complexity, non-linearity, and adaptability have been termed *Complex Adaptive Systems* (CAS, [1]).

The terms *chance*, *randomness* or *stochasticity* are different expressions related to the unpredictability of some events. Whether chance is a true feature in nature, as suggested by Prigogine [2], or simply the result of our inability to accurately observe and model natural phenomena is a matter of debate. However, the existence of *apparent* stochastic phenomena is undisputed. Throwing a dice or playing roulette are ways to produce a random outcome (otherwise there would be no game), despite the fact that these processes are believed to be ruled by the deterministic laws of Newtonian physics. In biology, the two pillars of the theory of evolution are selection and variation. The latter assumes randomness in the way DNA mutations and recombination take place. At a high level of biological organisation, the exact timing, location, and amplitude of extreme events such as pest outbreaks cannot be precisely predicted, although after they have occurred, their space-time evolution may be modelled statistically. These examples point to the central role of stochastic phenomena in real world physical and biological systems, and to the importance of chance in shaping the dynamics of such systems. Natural systems are therefore complex and adaptive systems partially controlled by stochastic phenomena, which makes them difficult to analyse and even harder to predict.

In his seminal work on ecosystem resilience, Holling [3] pointed to the incapability of conventional deterministic models to represent real world living systems because of their inability to integrate complexity, non-linearities, and stochasticity in an appropriate manner. Since Holling's contribution, developments in biological and ecological modelling have explicitly incorporated stochastic processes, although these have generally been built on deterministic *skeletons* [4]. The advent of individual based modelling has also led to population models directly built on stochastic processes occurring at the individual level [5]. Both types of approaches have shown the importance of stochastic processes on population dynamics, confirming the original insight of Holling. The theoretical and mathematical developments of stochastic models (or model components) of animal populations have greatly progressed, but this is not the case for ecosystem models in general and food-web dynamics models in particular. These still depend primarily on deterministic equations that relate predator species and their prey, though stochastic components are sometimes considered in addition to deterministic skeletons.

Apparent stochasticity in ecosystems may be the rule, but this does not mean that ecosystem dynamics are totally random. This is because *Nature*'s configurations are constrained by an ensemble of physical laws (e.g. gravitation, conservation of mass and energy) and evolutionary contingencies (e.g. pool of existing species, rate of genetic mutations). As a result, and as Cury et al. [6] point out, ‘*Nature may not be predictable but it is not totally unpredictable either*’ and it is the combination of stochasticity and constraints that drives the spatial and temporal dynamics of ecosystems. Given the importance of chance (stochasticity) and necessity (constraints), these two elements should have a central place in the development of ecosystem models.

A central issue in the modelling of food-web dynamics has been the use of functional responses that describe trophic functional relationships. The debate on the theoretical foundations for particular functional responses is still open [7], [8]. Deriving functional responses from empirical observations has proven difficult because trophic data that can be raised to the population level are parsimonious and empirical relationships are usually masked [9]. In addition, models of food-web dynamics are known to be highly sensitive to small variations in the shape of the functional responses [3], [10]. Even when stochastic predator-prey systems have been modelled, these rely on deterministic skeletons and therefore assume that the underlying relationship between prey and predators can be defined deterministically [11].

To our knowledge, only one modelling approach has escaped the deterministic formulation of functional responses. This work was published by Mullon et al. [12] and constitutes a novel alternative to existing food-web dynamics models. A major innovation in this work is the combination of the two fundamental ingredients: randomness (chance) and constraints (necessity). More precisely, in this model, trophic flows (the amount of prey eaten by a predator) are randomly chosen within a set of possible values that fulfil specific physical and biological constraints. The model structure is that of a Non-Deterministic Network Dynamics (NDND) model. Its design is general and can, in principle, be used to simulate the dynamics of any food-web or other similar networks [13]. Mullon et al. present a specific application for the Benguela ecosystem on the basis of which they conclude that “*this model reproduces in a robust manner observed patterns of variability and can be used to question the relevance of other modelling approaches of ecosystem dynamics with regard to determinism, constraints and stochasticity. Referring to a non-deterministic model without any functional relationships and environmental or anthropogenic forcing can help in avoiding misleading advice based on the belief that we can explain the causes of observed patterns, which may simply result from basic structural constraints within which the ecosystem functions*”. Despite the important implications of such conclusion for the research community working on ecosystem models, the work of Mullon et al. has received very little attention. Five years after it was published, the article by Mullon et al. has been cited once, and to date, no application of this model has been published for other areas. Does the model really work? Are the conclusions robust?

The objective of the present paper is to evaluate the replicability of the model presented by Mullon et al. For this purpose, we revisit the equations and input parameters that form the structure of the original model, which we term ‘MMM’ for ‘Mullon's Minimal Model’ and implement a comparable simulation model. In this contribution, we restate the model principles and provide a detailed account of the model structure, equations, and parameters following a model description protocol known as ODD (Overview, Design concepts, and Details, [14], [15]). We use this platform to simulate the dynamics of the Benguela food-web, the original case study of the MMM. We use these simulations to evaluate the replicability of the MMM and the conclusions reached by Mullon et al. in their original contribution.

## Model Formulation

The formulation of the non-deterministic network dynamics model for food-web is given below, following the ODD protocol [14], [15]. The ODD protocol was designed to provide detailed information about simulation models so that these can be made easier to understand and to duplicate. The protocol was originally designed for individual based models (IBMs), and several components of this framework are specific to IBMs. However, we found many of components of the ODD protocol well suited for the description of the NDND model and the sections below follow the suggested headings of the original ODD protocol. The present model sometimes departs from the original formulation of the MMM. When this is the case, we have highlighted the difference between the two models and presented the justification for such departure.

### Model purpose and principle

The purpose of the NDND model is to simulate food-web dynamics, i.e. the interannual fluctuations of the biomass of species and the trophic flows between them. The fundamental principle is that the flows of biomass between predators and prey are not deterministic, but are instead drawn randomly, given that they satisfy an ensemble of physical, physiological, and life-history constraints. Physical constraints are set by the law of conservation of mass, i.e. the total biomass in the food-web is maintained constant if the system is isolated (i.e. when there is no import, loss or export of biomass). Thus, fluctuations of total biomass in the food-web are solely the result of the balance between import of biomass into the food-web (e.g. new production) and export of biomass outside the food-web (e.g. fishing, egestion and metabolic losses). This is a so-called mass-balanced model and, as such, it shares similarities with other mass-balanced models like Ecopath with Ecosim (EwE, [16], [17]). Physiological constraints are set by the maximum rates of ingestion by individual organisms of a given species, where this upper limit (i.e., the maximum *consumption over biomass* ratio) is termed *satiation*. In addition, the maximum growth and mortality rates of a population are limited as a function of the species' lifespan, where populations of short-lived species fluctuate at potentially higher rates than populations of long-lived species. The relationship between life span and population growth and mortality rates has both theoretical and empirical support [18], [19].

The result is a mass balance model in which 1) trophic flows are drawn randomly for all species, 2) ingestion never exceeds satiation, and 3) the rate of biomass variation is bounded. These principles are identical to those of the MMM. We complemented the principle that the minimum biomass attainable by a population is greater than zero and corresponds to a ‘refuge’ biomass below which the species is no longer accessible to predators.

We present below the mathematical formulation of the model dynamic equation and constraints, as well as the definition of the model input parameters and how these can be related to ecologically meaningful quantities.

### Entities, state variables, and scales

The structuring elements of the model are trophospecies and trophic interactions, which together constitute a food-web topology. A trophospecies represents an ensemble of organisms that share the same set of trophic interactions. All individuals in a trophospecies may not belong to the same taxonomic species; in the following text, we use the word *species* to mean trophospecies. Trophic interactions indicate the possible transfer of mass between two species. Contrary to food-web structure models, which are concerned with how food-webs assemble and how their structure can evolve with speciation and extinctions, the food-web topology of the NDND is fixed and provided as input data. The indices, variables, parameters, constraints, and master equation of the NDND are summarised in Appendix S1, alongside the original formulation of the MMM and their equivalence in the Ecopath with Ecosim framework (EwE, [17]).

*State variables*. The following variables are required to fully define the state of the system at any time: biomass of individual species *i* (*B_i_*), trophic fluxes, i.e. biomass flux from species *i* to species *j* (*F_ij_*), and import (*I_i_*) and export (*E_i_*) of biomass of individual species *i* in or out of the system.

*Input parameters* consist of the following:

- assimilation efficiency (*γ_i_*): the proportion of biomass ingested by species *i* that can contribute to growth, reproduction, and maintenance (0<*γ_i_*<1),
- satiation (*σ_i_*): the maximum consumption rate by species *i*, expressed as a proportion of the current biomass of species *i* (*σ_i_*>0),
- inertia (*ρ_i_*): the minimum and maximum rates of biomass change are set respectively to 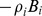 and 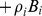 (*ρ_i_*>0),
- metabolic and other losses (*µ_i_*): The quantity *µ_i_* represents the rate of biomass loss through metabolism, e.g. locomotion and maintenance, and ‘other’ mortality (mortality other than the predation explicitly represented in the model) (*µ_i_*>0),
- Refuge biomass (*β_i_*): the irreducible biomass of a species (*β_i_*>0).

### Scales

The temporal resolution of the model is annual, i.e. each time-step in a simulation is one year. The spatial scale is a large ‘self contained’ ecosystem. The ecological scale is a ‘food-web’, typically from primary producers to top predators. In the present study, we have applied the model to the simplified Benguela ecosystem as defined in Mullon et al. [12].

### Process overview and scheduling

The NDND model represents variations in the biomass of individual species as the result of import, export, and trophic interaction terms. The food-web dynamics is fully defined by the equation that describes variation in the biomass of individual species. This master equation, in continuous time, is:
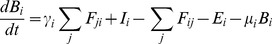
(1)


For simulation purpose, the NDND operates in discrete time steps, with one-year time intervals. Assuming that trophic flows, imports, and exports are constant during the integration period, the discretised form of the master equation can be derived (details of this derivation are presented in Appendix S2):
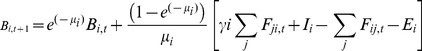
(2)


In equation 2, the term 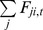 represents the total biomass of prey species consumed by species *i* between two consecutive time steps; the term 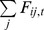 represents the total biomass of species *i* consumed by its predators during the same period.

Contrary to standard food-web models, the trophic flows are not defined by a deterministic equation. Instead, they are drawn from the set of possible flows which satisfy the following constraints:

-flows are possible (i.e. species *j* is a predator of species *i*),

-flows are positive:

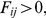
(3)

-resulting biomasses are not below ‘refuge level’:

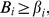
(4)

-change in biomass is constrained by inertia:

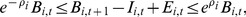
(5)

-total food intake is limited by satiation:

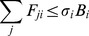
(6)At each time step, a vector of random flows (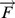), which satisfies all the above constraints, is drawn. Solving the multiple constraints equation is a complex computational problem and the solution employed here is presented in details in Subbey et al. (Subbey S, Planque B, Lindstrøm U, submitted. Exploring stochasticity and imprecise knowledge based on linear inequality constraints. SIAM Journal on Scientific Computing).

### Design concepts

The main design concept of the model is a food-web topology (species and their trophic links) dynamically modelled by the combination of chance and necessity; chance being modelled by a stochastic process (the random drawing of trophic flows) and necessity being expressed by the limited set of physical, physiological and life-history constraints outlined above.

### Emergence

There are several emerging properties that can be expected from the model, several of which were explored with the MMM. These include:

- Temporal dynamics of individual species, e.g. temporal autocorrelation, quasi-cycles, abrupt shifts,
- Temporal dynamics of integrated food-web properties, e.g. total biomass, total trophic flows, total assimilation efficiency, mean trophic level,
- Diet fraction, i.e. the percentage of various prey consumed by a predator,
- Stock-recruitment relationships,
- Trophic regulations, e.g. top-down *vs* bottom-up controls measured as the temporal correlation between biomass of predators and prey,
- Predator-prey functional responses.

Many of the above quantities can be derived from field or experimental data and therefore constitute an ensemble of criteria against which model results can be evaluated. The above list is not exhaustive and creative researchers will surely find other ways to describe additional emerging properties of the system.

### Adaptation, individual level properties, and collectives

Adaptation is not included in the model design. The topology of the food-web and model parameters are set and the model does not include adaptive mechanism by which these might change. The structuring elements of the model are trophospecies and properties at the level of individual organisms are irrelevant, as are collectives.

### Interactions

The only interactions in this model are trophic interactions, i.e. flow of biomass between predator and prey species.

### Stochasticity

Randomness is central to the NDND model since the key elements, i.e. the trophic flows between species, are drawn randomly from a set of possible flows. In essence the model is tychastic [20], i.e. it is concerned with the subset of all possible transitions from one time step to the next. In practice, the simulations are stochastic, i.e. only one combination of trophic flows is drawn from the set of possible ones.

### Initialisation and input parameter values

The structure of the food-web (i.e. the food-web topology defined by the list of species and possible links between them) must be set. In addition, NDND requires initial values for individual species biomass (*B_i,t0_*), and the five species-specific parameters (*γ_i_*, *σ_i_*, *ρ_i_*,* µ_i_* and *β_i_*).

### Input data

Species-specific data is required for import (*I_i_*) and export (*E_i_*) to express flows to and from the model domain. These can be invariant over time or variable at each time step.

### Departure from the original MMM model

We have tried to keep the formulation of the NDND food-web model as close as possible to the original formulation of the MMM. There are however some differences. These are as follows:

- *Discrete formulation of the master equation:* In the MMM, the main equation was presented for populations at equilibrium (eq 2 in Mullon et al.) and can be used to express the dynamics of the system in continuous time. The continuous form of the master equation of the NDND model is presented above (eq. 1) and is very close to the one presented in the MMM (Appendix S1, but see below the difference concerning the import term). In addition, we provide the discrete form of the master equation, which is necessary for iterative computation of the model (eq. 2). The conversion of the continuous equation (eq. 1) into its discrete analogue (eq. 2) is presented in Appendix S2.
- *Refuge biomass* (*β_i_*). This was not a feature of the MMM, in which biomasses were only constrained to remain positive. We found that this could lead to situations where a species would reach extremely small biomass and therefore flows to and from this species would be very small in comparison with other flows. This could result in computational problems due to scaling difficulties. Modelled biomass levels could fall below the weight of a single individual, which is biologically implausible. The combination of very low biomass with bounded growth rates could also lead to unrealistically long recovery times. The introduction of the additional parameter *β* for refuge biomass in the NDND solves these problems. It also introduces the possibility to model actual refuge strategies for species which can become inaccessible to predation at low densities. For the current simulations, the refuge biomass levels were arbitrarily set to 1% of the starting biomass levels.
- *Inertia*: In the MMM, inertia was expressed in such way that the maximum potential increase and decrease in species biomasses were equal. As a result species biomass could decline at much faster rates than they could recover. For example, in the MMM, zooplankton biomass could vary by up to 85%. This implied that the population could decline by 85% in one year, to reach 15% of its original biomass. However, to grow back to its original biomass at a maximum increase of 85% every year would require about 3 years (reaching 28% of the original biomass in year 1, 51% in year 2 and ≈100% in year 3). We used an alternative formulation for inertia to avoid such asymmetry between the rate of biomass decline and recovery. Instead of setting maximum biomass increase and decrease to be equal, it is the minimum and maximum rates of biomass change that are equal in the NDND (i.e. respectively 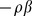 and 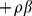). This is presented in more details in Appendix S3.
- *Import and assimilation terms*: In the MMM, the import term *I* strictly referred to inflow of nutrients to autotroph species (e.g. phytoplankton). For those species, the term *γ* did not refer to assimilation efficiency (as it did for other species), but to conversion efficiency from nutrient to biomass. This was confusing because the term *γ* could refer to two different biological processes: 1) conversion of nutrients into species biomass through photosynthesis, when it is applied to *I*, and 2) assimilation efficiency of ingested food otherwise. We also found this notation restrictive because it is only possible in the MMM to use the term *I* for import to autotroph species, but not to heterotroph or mixotroph species. In the NDND model, *I* represents the direct import of biomass, either through new production (autotrophs, mixotrophs) or through migration and transport. In the case of production, *I* represents the converted biomass and there is no longer need for the conversion efficiency. In the present model, the term *γ* is only used to describe assimilation efficiency.
- *Sampling*: In the MMM, at each time step, the biomasses of all species were randomly drawn from the set of possible solutions that satisfied the model constraints using linear programming. This approach can be viewed as the inverse problem of determining a set of flows, which result in the drawn biomasses. The approach in NDND, however, involves solving the forward problem of determining an ensemble of vectors of flows that satisfies a set of constraints and the use of flows to determine future biomasses. Viewed in terms of probabilities, the MMM approach considers all possible future states (biomass vectors) to be equiprobable, while in the current model, all possible transitions (flow vectors) have equal probability.

We have provided above an update of the original formulation of the MMM and a detailed description of the model state variables, input parameters, and constraints. We provide in the supplementary material the correspondence between state variables and input parameters in the NDND model and their formulation in the MMM as well as in the EwE context (Appendix S1). The implementation of the model was done in Matlab.

## Results

### Reproduction of the MMM simulations

**Figure 1:**
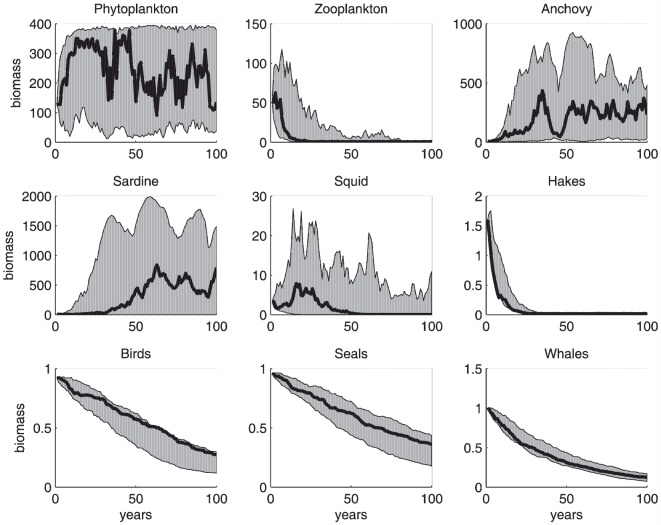
Fifty simulations of the NDND for the Benguela ecosystem, using initial biomass, input parameters and food-web topology as in the original MMM paper. The black thick lines show biomass trajectories for an individual simulation. The grey areas indicate the ranges of biomass values covered by the 50 simulations.

We ran 50 simulations of the Benguela ecosystem dynamics using the same set of parameters as in the MMM over a period of 100 years. The simulations produced time series of individual species, but failed to convincingly reproduce the ecosystem dynamics presented in the original study. In particular, the modelled populations of zooplankton, hakes, birds, seals, and whales declined to low or very low levels, whilst anchovy and sardine populations reached levels about one to two orders of magnitude greater than those simulated in the MMM or observed in the wild. These features were consistently observed in the 50 simulations (Figure 1).

### Parametrisation of the Benguela ecosystem model

**Figure 2:**
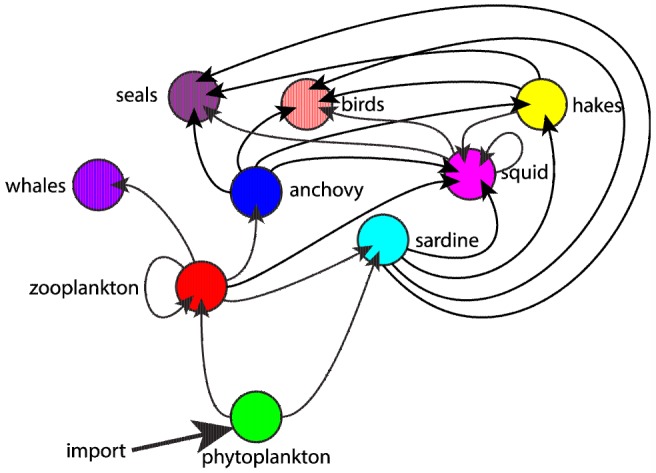
The food-web topology of the modelled Benguela ecosystem. Arrows indicate a trophic relationship and point towards predators. The primary production is set by a fixed annual import of phytoplankton.

Since the initial model configuration did not convincingly produce patterns of variability similar to those of the MMM, we explored if other model parameterisations could produce realistic patterns similar to those presented in the original study. This was done by trial and error, on the basis of the original food-web modelling study by Shannon et al. [21]. We kept the original values for the ‘other’ mortality coefficient (*µ* and derived the inertia coefficient (*ρ*) from the original values, following the equations provided in appendices 1 and 3. The initial biomasses were set by trial and error and the refuge biomasses (*β*) were set to 1% of the starting biomass values. The derivation of assimilation efficiency coefficients in the MMM was unclear. Assimilation efficiency is likely to vary within and between species because it depends on food quality; generally, carnivores have higher assimilation efficiencies than herbivores. This is now reflected in the use of assimilation efficiency (γ) derived from the work of Yodzis and Innes [22]. The satiation coefficient was derived from *µ* and *γ* to ensure that maximum feeding rates were greater than the requirements for species maintenance under absence of predation. These coefficients were then adjusted by trial-and-error. Model parameters in the revised configuration are given in Table 1. The topology of the food-web remained unchanged except for anchovy, which was changed to only feed on zooplankton (Figure 2), while it also fed on phytoplankton in the MMM. This seemed unrealistic given that bite-feeding, rather than filter-feeding, is the dominant or exclusive pattern of anchovy feeding [23], [24]. Despite the corrections above, the model structure (topology) and parametrisation was not satisfactory because some key components of the ecosystem were missing in comparison with the original study of Shannon et al. For example, the MMM does not include meso-pelagic or benthic species that play an important role in the energy transfer of the Benguela system. In such a situation, one should not expect that the food-web model could represent realistic dynamics and biomass levels for all species simultaneously. As in the original study, we ran 50 simulations of the model with the revised structure and parameters. The outputs are presented for years of simulation 101 to 200, to avoid patterns eventually driven by initial biomass conditions.

**Table 1:** Input parameters of the Benguela pelagic ecosystem model.

Species	Import (t.km^−2^)	Initial Biomass (t.km^−2^)	Assimilation efficiency (*γ*)	Other losses (*µ*)	Inertia (*ρ*)	Satiation (*σ*)	Refuge Biomass (*β*t.km^−2^)
Phytoplankton	700	25.00	-	15	0.683	-	0.250
Zooplankton	-	2.00	0.45	12	0.615	80.0	0.200
Anchovy	-	10.21	0.85	1	0.470	15.1	0.102
Sardine	-	10.59	0.50	1	0.405	6.0	0.105
Squid	-	0.70	0.85	1	0.531	4.7	0.001
Hake	-	2.00	0.85	1	0.300	4.7	0.002
Birds	-	0.20	0.85	10	0.113	15.3	0.001
Seals	-	0.53	0.85	8	0.095	12.2	0.001
Whales	-	0.10	0.85	9	0.049	13.8	0.001

### Reproduction of pseudo-cycles

**Figure 3:**
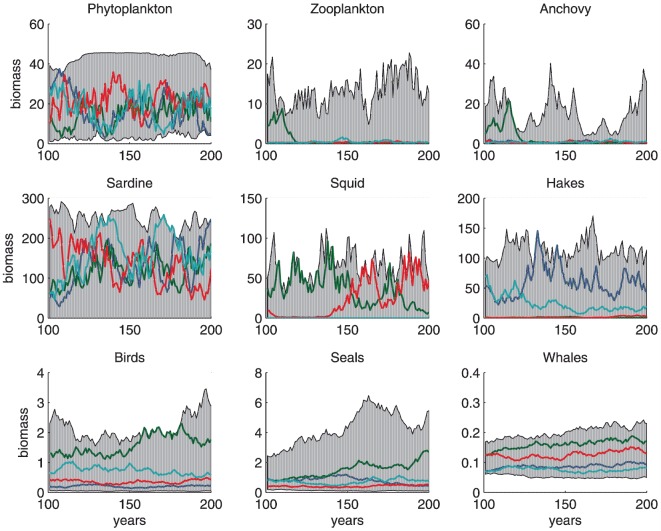
Fifty simulations of the NDND for the Benguela ecosystem, using revised initial biomass, input parameters, and food-web topology. The coloured lines show biomass trajectories for four individual simulations. The grey areas indicate the ranges of biomass values covered by the 50 simulations.

**Figure 4:**
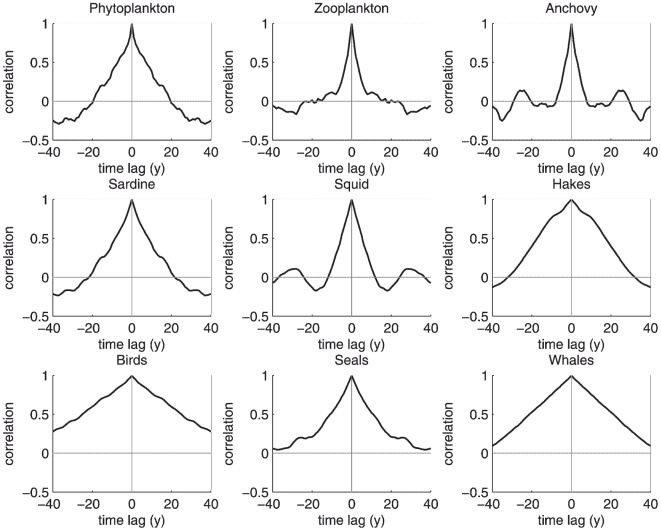
Autocorrelation series for a given run (time steps of the simulation on the X-axis; correlation coefficient on the Y-axis). An estimation of the cycle duration is provided by the lag associated with positive peaks, or twice the lag with negative peaks.

**Figure 5:**
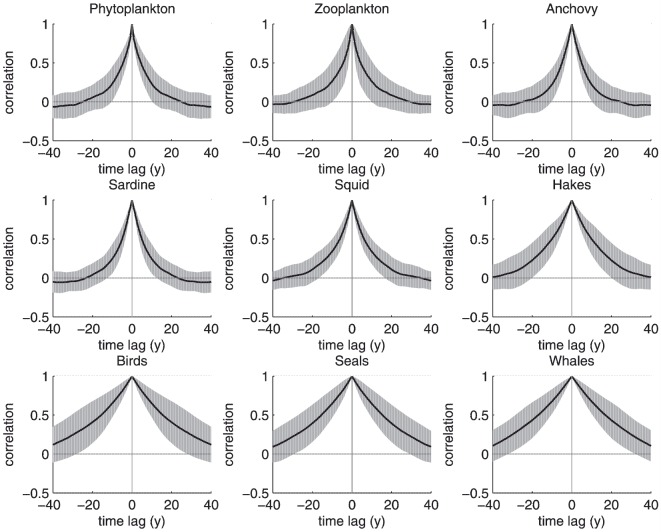
Mean (central line) and SD (shadow area, mean ± SD) on each side of the central line of autocorrelations for a model experiment of 50 runs (same axis as in Fig. 4).

Individual simulations were uncorrelated, as expected, given the stochastic nature of the model (Figure 3). For all species, the simulated biomasses covered a large range, sometimes spanning several orders of magnitude. Year-to-year and decadal variations were evident from the simulations. As in the MMM, the model appeared to produce series with pseudo-cycles. It was not as clear in our simulations that these were predominantly seen at intermediate trophic levels. We also performed an analysis of the autocorrelation of the simulated abundance series (Figure 4). We found that the autocorrelation functions could vary considerably and that the cyclic patterns and trends could substantially differ between the different simulations. The values of autocorrelation coefficients averaged over the 50 runs indicated an increase in the length of pseudo-cycles (or a dominance of trends) with increasing life span, but not necessarily with trophic level (Figure 5). Like Mullon et al., we conclude that, for a given species, the length of its cycle is highly variable and that the pattern of pseudocycles of irregular lengths can be related to the species life span.

### Reproduction of other patterns of variability

**Figure 6:**
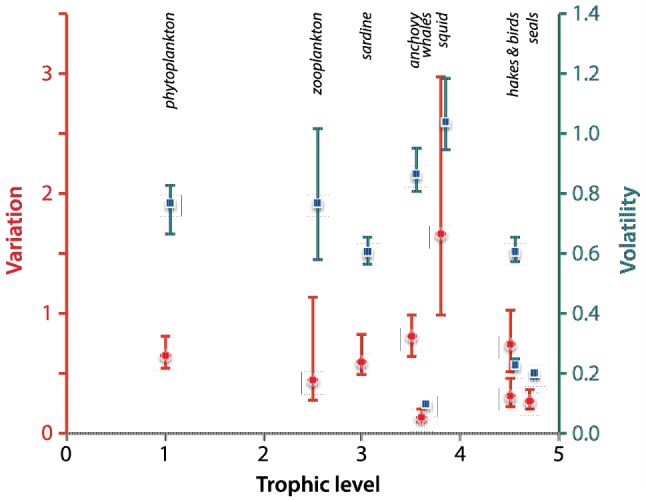
Variability patterns in the simulated ecosystem: values of variation (red circles) and volatility (blue squares) as a function of trophic level. Central points indicate median value and error bars indicate the interquartile ranges for the 50 simulations.

In the MMM paper, other patterns of variability were quantified by considering the variation and volatility of abundance series. We followed the same approach. Variation was measured as the ratio of interquartile range to the median of the series and volatility was measured as the ratio between the range of observed values in a given period and the value central to this period, which represents the ratio of short- to long-term variation. We found that variation and volatility had highest values for squid, sardine, anchovy, and zooplankton (Figure 6). In our simulations, the relationship between variation, volatility, and trophic levels was not clearly apparent, although top predators (hakes, birds, seals, and whales) displayed the lowest variability. Unlike Mullon et al., we cannot conclude that the indices of variation and volatility indicated a dome-shaped pattern, with highest values at intermediate trophic levels, which was interpreted as a simple illustration of a wasp-waisted system, *sensu* Bakun [25].

### Reproduction of diet patterns

**Figure 7:**
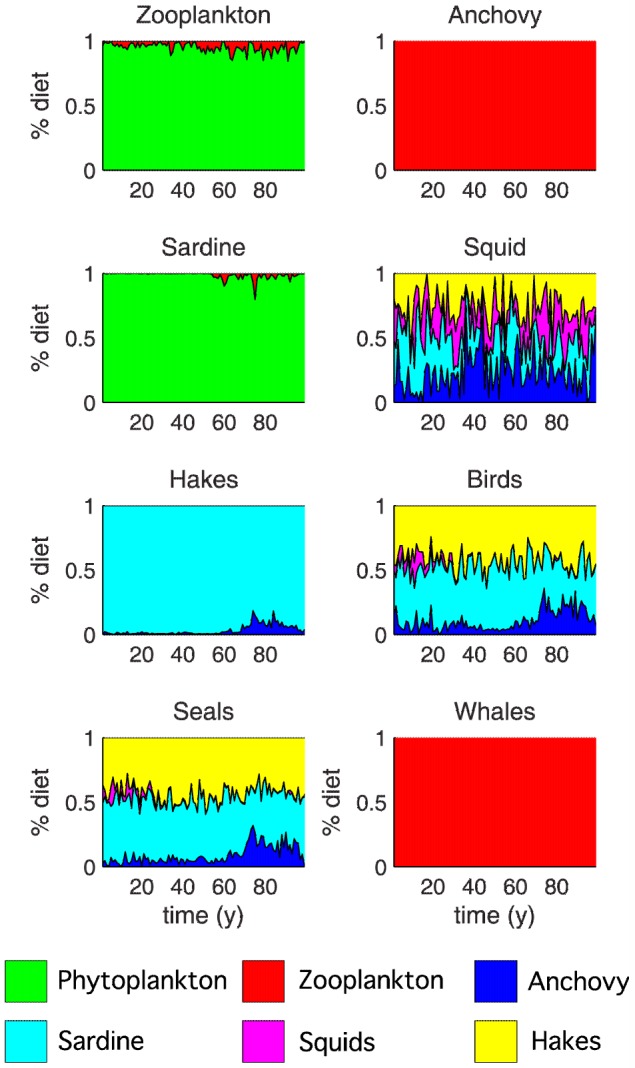
Diet composition dynamics (one single run of 100 years) for all model compartments except phytoplankton. This figure also shows the connectivity of the network and its variability in time.

The list of prey species that determine the diet of a predator was defined by the food-web topology (Figure 2). However, how the proportions of various prey vary in time was determined by the relative abundances of all prey and predators in the food-web and by the stochastic process from which random trophic flows were drawn at each time step. For some species (e.g. squid), diet composition varied substantially from year-to-year, whilst for others (e.g. seals), it remained relatively stable (Figure 7). Interestingly, the diet of squid presented in the MMM was dominated by zooplankton, with minor predation on sardine and anchovy, but in our simulation, zooplankton was virtually absent from squid diet, which was mainly composed of anchovy, sardine, squid, and hakes in highly variable proportions. This variation in diet between the two models illustrated how model configuration and stochastic simulations can lead to a wide range of system configurations.

**Figure 8:**
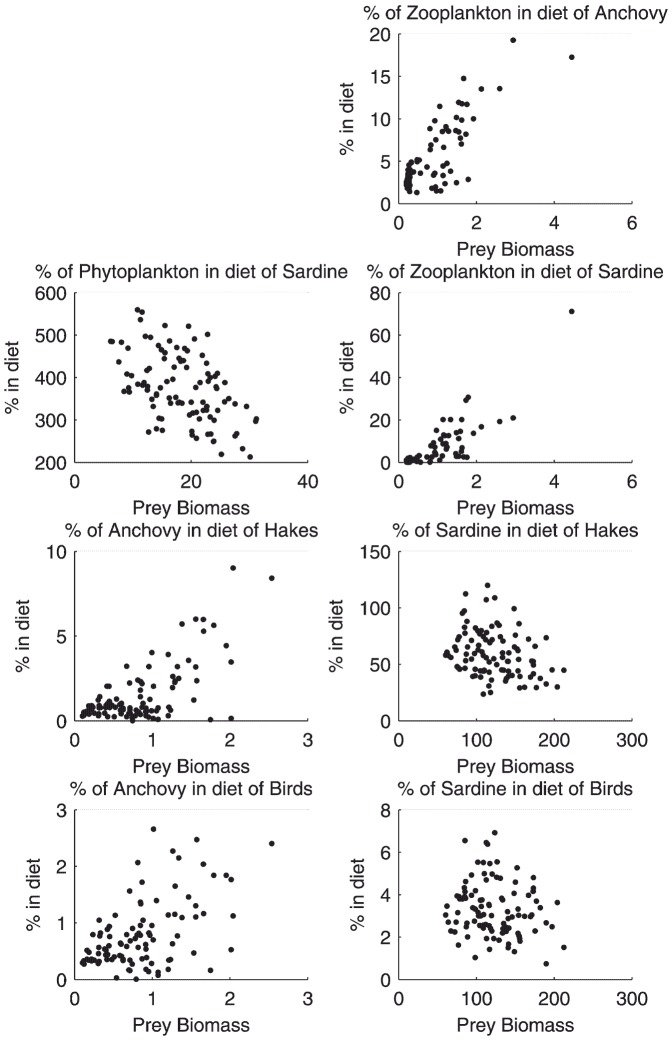
Simulated diet/abundance relationships. Captions indicate the name of predator species followed by the name of one of the major prey species for a predator. The **Y**-axis represents the proportion of prey in the predator diet and the **X**-axis represents the abundance of the prey species.

Trophic functional relationships, which are formally excluded from the model structure, can be investigated as emerging properties. From the single simulation represented in Figure 8, we observed three types of configurations: positive relationship (e.g. hake feeding on anchovy), negative relationship (e.g. sardine feeding on phytoplankton), and absence of relationships (e.g. birds feeding on sardine). Even in the case of apparent relationships, there was a large scattering of the simulated data, which reflected diet variations that have been observed in the field through the analysis of stomach contents of predators in relation to prey abundance [26], [27].

### Reproduction of stock-recruitment relationships

**Figure 9:**
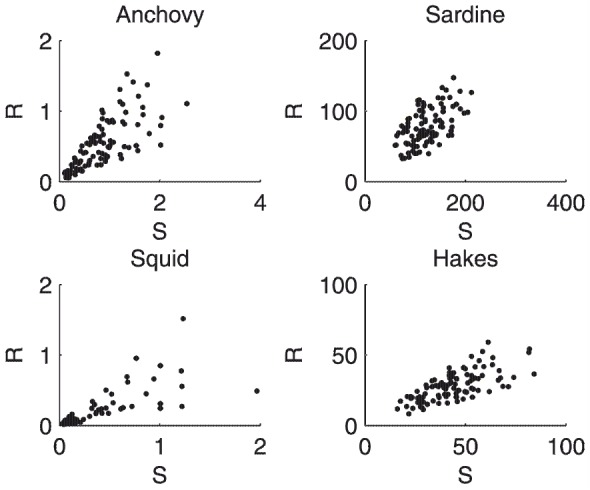
Examples of stock-recruitment (S, R) relationships using the variation from population biomass minus annual metabolic losses to new population biomass as a proxy for recruitment: 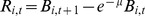.

We defined a proxy for recruitment, expressed as the variation in population biomass corrected for losses due to metabolic activities and other losses: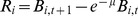 (note that this recruitment equation is different from that used in the MMM paper). The resulting stock-recruitment plots (Figure 9) displayed noisy linear positive relationships. Here, the stock-recruitment relationship emerged as a consequence of ecosystem functioning, not as a causal principle. The simulated patterns mimicked published data, although we saw no clear sign of density dependence, unlike in the MMM and as is generally assumed in fisheries stock recruitment models (e.g. Beverton–Holt and Ricker funtions) [28].

### Reproduction of interdecadal variations in trophic interactions

**Figure 10:**
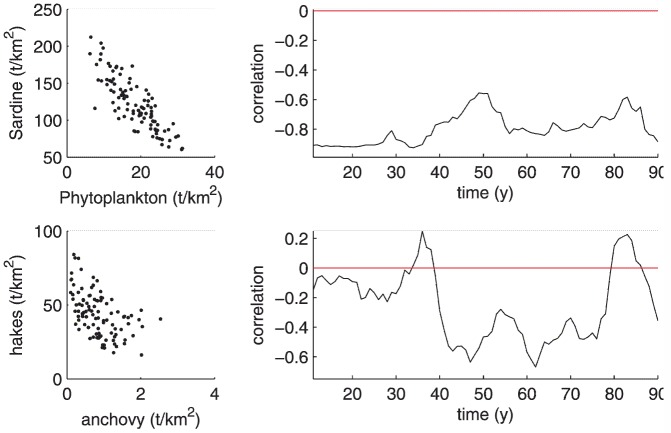
Left: Biomass of predator versus biomass of prey over 100 y for sardine-phytoplankton (top) and hakes-anchovy (bottom). Right: sliding correlations between predator and prey biomass. The correlation is calculated over a 20 y window. These highlight the general relationship between prey and predator (top-down vs. bottom-up) and the interdecadal variations in trophic controls.

Interactions between prey and predator in marine systems can lead to apparent correlations between biomass time series. When the correlation is negative, the relationship is generally described as being top-down controlled (the predator controlling the abundance of the prey), and when it is positive, the prey-predator system is said to be bottom-up controlled (the prey controlling the abundance of the predator) [29]. Although simplistic, this description of the trophic controls between predator and prey is easy to construct from field data. These trophic controls have been shown to fluctuate at interdecadal time scales (see e.g. [30]). The modelled dynamics highlighted the strong negative correlation between sardine and phytoplankton over the 100 y simulation period, indicating that the standing stock of phytoplankton was controlled by grazing from sardine (Figure 10). The relationship between hake and anchovy was also negative, but large fluctuations appeared on a decadal time-scale, which occasionally resulted in positive correlations) thereby mimicking interdecadal fluctuations observed in real systems.

## Discussion

### Replicability of the original model

The original study of Mullon et al. used a novel non-deterministic modelling approach to simulate the dynamics of marine food-webs. In this way, the ‘null model’ could serve as a reference for other deterministic models. Based on this approach, Mullon et al. reached important conclusions regarding the dynamics of food-webs when modelled with only few assumptions and without explicit deterministic formulations of trophic functional relationships. Here, we have tried to reproduce the original model structure and dynamics and test whether the conclusions reached in the original study were still valid in our replication.

We found that when we used the input parameters from the original model, we could not reproduce the food-web dynamics presented in the original study. This is surprising given that the equations of the two models are identical except for the constraints on inertia and refuge biomass. Our revision of these equations should have led to a more stable system with a lower probability of rapid population collapse. It is not clear how input parameters and starting biomasses presented in the MMM paper were derived from the original modelling study of Shannon et al. [21], so we went back to the original study to revise these values, using input from other sources to document assimilation efficiencies [22]. Even with these revisions, we had to perform trial-and-error runs until we could produce a ‘realistic’ set of simulations for the Benguela pelagic food-web dynamics.

The major conclusions from the MMM paper concern the reproduction of several ecosystem dynamic patterns: pseudo-cycles, variation and volatility, diet, stock-recruitment relationships, and correlations between species biomass series. For all of these aspects, we reached similar conclusions, although these were often not as strongly supported as suggested in the original study. We found pseudo-cycles of various periods for the different species in the food-web and that variation and volatility varied between species and were lower for higher trophic levels. Noisy trophic functional relationships emerged from our model simulations and large year-to-year fluctuations in diet composition could happen for intermediate predators with a range of potential prey. Simulations also indicated that noisy stock-recruitment relationships were possible. We also found correlations between species biomass series, which could either be stable or vary at interdecadal time-scales. Despite differences between the original MMM and the present model, we show that a non-deterministic model *with a minimum set of constraints* can recreate important ecosystem dynamical patterns. This result is crucial, in particular, because deterministic models that require a large amount of assumptions and input parameters generally fail to reproduce most of the patterns described above. For example, EwE models, which include explicit trophic functional responses and are fitted to historical data, usually fail to produce realistic year-to-year variability in population biomass when projected forward, but instead generate smooth patterns over longer time scales (e.g., [31]). In addition, most food-web models, including EwE, are highly sensitive to assumptions and data uncertainties regarding trophic functional relationships [10], [17], [32], a problem that is absent from the current modelling approach, in which such relationships are not an input to the model but emerging properties of the system.

### Ecosystem variability

In several instances during our trial runs, the food-web state could be trapped, i.e. it was not possible to find a combination of trophic flows that would satisfy all constraints. Our simulations also appeared more variable than in the original study. Since the NDND has an additional constraint (refuge biomass) and the constraint on inertia is stronger (see Appendix S3), the most plausible explanation for the increased variability lies in the method used to generate the random transition from one food-web state to the next. There are fundamental differences in the two approaches. The inverse problem of determining flows given biomasses should, in principle, not result in a unique solution (there may be an infinite combination of flow configurations for the same observed biomass). The linear programming approach adopted in MMM limits the solution space to flow values at the vertices of the polytope defining the constraints. In the NDND approach, however, the vertices are a subset of the solution space, which also includes the interior points of the polytope. Hence, sampling in the case of NDND is expected to show larger variability than with the MMM. Further, by the nature of the sampling procedure, it is possible to generate realisations, which though mathematically right (i.e. satisfying the constraints on the flow functions) are biologically implausible. Variability in the model may exceed what is observed in the wild. If this is the case, it would mean that the set of constraints currently used is insufficient to restrict model variability within observable limits and additional constraints may be necessary. This would require further investigation and comparisons with real systems.

### Model complexity

The model configuration for the Benguela is a simple one, with only nine trophospecies and 20 trophic links, so the computational problem is both relatively simple (a polytope of 20 dimensions to explore) and fast (<0.1 s per year of simulation). However, more complex food-web models, such as those constructed in EwE containing >50 species and several hundred links will likely lead to computational challenges. For instance, the simplest case of uniform sampling of a convex polytope in high dimensions leads theoretically to what is termed an NP-hard problem (i.e., belonging to the class of problems that are, informally, "at least as hard as the hardest problems in Non-deterministic Polynomial-time", see e.g. [33]). Since the number of constraints is finite, a complete characterization of the polytope could be argued to be known. The problem therefore reduces to the task of finding fast algorithms (usually deterministic) that perform exhaustive enumeration on a convex polytope. Unfortunately, deterministic algorithms that perform can be infeasible in high dimensions since the number of such vertices could scale exponentially with the dimension [34]. An alternative approach to the deterministic approach is to use stochastic algorithms (Monte Carlo based with acceptance-rejection rules) to sample the polytope. However, even the fastest of such algorithms is known to suffer scaling problems (with respect to mixing time, see e.g. [35]) in high dimensions, especially when the polytope is highly heterogeneous, as in the case of our food-web model. Fortunately, to investigate the dynamics of whole food-webs (i.e. rather than the dynamics of individual taxa), such levels of food-web complexity are often not neccessary and an approach based on the use of few, well defined trophospecies groups might capture most the food-web properties [36].

### Model improvements

The NDND framework is a potentially powerful framework to investigate food-web dynamical patterns that are driven by a few sets of constraints and can therefore be used as a reference model against which more complicated models can be evaluated, as originally suggested by Mullon et al. However, several developments appear necessary for such a model to become a general and powerful tool in the study of ecosystem dynamics. Setting the values of the model input parameters is a difficult task for which there is yet no objective and transparent methodology. This is no surprise, given the complexity of the task. It took nearly 20 years before such method became available for Ecopath models [37]. Hopefully methods used for parameter optimization in Ecopath can be reformulated for the NDND models, so that setting model parameters will be less based on trial and error and more on ecological theory and available field data. Using metabolic theory of ecology [38] to estimate metabolic losses and maximum consumption rates (satiation) and life-history theory to derive growth rate (inertia) estimates will also make the model more general and easier to parameterise.

The evaluation of model performance was not properly addressed in the MMM and this limitation remains in the current model. Since these are stochastic models which are not required to fit data in the conventional way (i.e. by fitting time series of biomass for example) there is, as of yet, no simple and accessible methodology to evaluate model performance or to perform sensitivity tests. Approaches to this problem may be rather different from the conventional techniques favoured by ecological modellers today (e.g., [39], [40]) and would likely involve a pattern oriented approach as advocated by Grimm and colleagues [41]–[43] combined with dedicated statistical inference for stochastic models [44] and hierarchical model evaluation techniques [45].

The original form of the MMM and the current form of the NDND model are prototypes and clearly, these models do not benefit from the experience of other species-based or size-based ecosystem models [32]. In the present contribution and the associated appendices, we have detailed as much as possible the hypotheses, equations, and computational aspects of the NDND to allow other researchers to test this approach and contribute to the model development. This should allow for the use of the NDND as a null model for comparison with other deterministic modelling approaches.

In its current form, the NDND simulates the dynamics of simple food-webs over annual time steps and in a single area. However, the mathematical formulation and computation can readily allow inclusion of spatialised food-webs (in a way similar to Ecospace or GADGET models [46], [47]), shorter simulation time-steps (seasons, months, days), or the modelling of age-structured populations.

## Conclusions

While randomness is generally considered a source of uncertainty for deterministic models, the current study supports the original conclusions of Mullon et al. that stochasticity can play a structural and central role in shaping key features of food-webs. In this, the NDND model shares similarities with models issued from the ecological neutral theory, which can also reproduce ecological patterns on the basis of few assumptions combined with stochasticity [48]. We do not claim that real systems resemble stochastic food-webs, but rather that the NDND approach can improve our understanding by making simplifying assumptions about complex systems.

The approach proposed by Mullon et al. [12], which combines chance (randomness) and necessity (constraints), is unique in the field of ecosystem modelling, although these two elements have long been recognised as shaping biological systems [49]. Their original conclusion, i.e. that *observed patterns of ecosystem variability may simply result from basic structural constraints within which the ecosystem functions*, is of great importance to ecosystem modellers and those who may use model outputs as a support for management decisions. These conclusions are supported, to a large extent, by the current replication of the model. However, model parameterisation and computational aspects remain difficult and these need to be investigated further. Hopefully, the present contribution will make this approach available to a larger research community and will promote the use of NDND as ‘null models of food-webs' as originally advocated.

## Supporting Information

Appendix S1 **Table presenting the main elements of the MMM, the NDND and the Ecopath with Ecosim models.** The table provides a comparative description of the indices, state variables, input parameters, constraints and equations for the three modeling approaches.
(PDF)

Appendix S2 **Integration of the model master equation in continuous form (eq. 1) into its discrete form, for time step** ***t*** **to time step** ***t+1*** **(eq. 2).**
(PDF)

Appendix S3 **Comparison of the variance in rate of biomass change in the MMM and NDND models as a result of distinct formulation of the inertia constraint (** ***ρ*** **).**
(PDF)
