# Supplementary material for: Non-Deterministic Modelling of Food-Web Dynamics
Source: PLoS One. 2014 Oct 9;9(10):e108243. doi: 10.1371/journal.pone.0108243 (PMC4191973; doi:10.1371/journal.pone.0108243)
Supplement: Appendix S1 — Table presenting the main elements of the MMM, the NDND and the Ecopath with Ecosim models. The table provides a comparative description of the indices, state variables, input parameters, constraints and equations for the three modeling approaches. (PDF) [file pone.0108243.s001.pdf]

Appendix 1. Comparison of the model elements in Mullon et al (2009), the NDND model and the Ecopath with Ecosim (EwE).

	**Mullon et al. (2009)**	**Non Deterministic Network Dynamics model**	**Ecopath with Ecosim**	**Units (NDND) / Comments**
Indices				
species	*s, r*	*i, j*	*i, j*	
time	*t*	*t*	*t*	y
State variable				
Biomass	B_S	B_i	B_i	$t.km^{-2}$
Flows	X_{SR}, X_{RS}	F_{ij}, F_{ji}	Q_{ij}, Q_{ji}	$t.km^{-2}.y^{-1}$
Import	I_S	I_i	I_i	$t.km^{-2}.y^{-1}$
Export	Y_S	E_i	$(F_i+e_i)B_i$	$t.km^{-2}.y^{-1}$ / F is fishing rate and e is emigration rate in EwE.
Parameters				
Assimilation efficiency	γ_S	γ_i	g_i	%
Satiation	σ_S	σ_i	Q_{max}	%
Inertia	ρ_S	ρ_i	-	$\rho_i \approx log(1+\rho_s)$
Other losses	μ_S	μ_i	$M_i = \frac{P_i}{B_i}(1-EE_i)$	% / P is Production and EE is ecotrophic efficiency in EwE
Refuge biomass	–	β_i	-	$t.km^{-2}$ / not used in Mullon et al.
Constraints				
Inertia	$\lvert B_{s,t+1} - B_{s,t} \rvert \leq \rho_s B_{s,t}$	$e^{-\rho_i} B_{i,t} \leq B_{i,t+1} - (I_{i,t} - E_{i,t}) \leq e^{\rho_i} B_{i,t}$	-	
Satiation	$\sum_r X_{rs} \leq \sigma_s B_s$	$\sum_j F_{ji,t} \leq \sigma_i B_{i,t}$	-	
Positive flows	$X_{rs} \geq 0$	$F_{ij} \geq 0$	-	
Minimum Biomass	$B_s > 0$	$B_i > \beta_i$	-	
Master equations				
Continuous	$\frac{dB_s}{dt} = \gamma_s\left(\sum_r X_{rs} + I_s\right) - \sum_r X_{sr} - Y_s - \mu_s B_s$	$\frac{dB_i}{dt} = \gamma_i \sum_j F_{ji} + I_i - \sum_j F_{ij} - E_i - \mu_i B_i$	$\frac{dB_i}{dt} = g_i \sum_j Q_{ji} - \sum_j Q_{ij} + I_i - (M_i + F_i + e_i)B_i$	
Discrete between t and t+1	-	$B_{i,t+1} = e^{(-\mu_i)} B_t + \frac{\left(1 - e^{(-\mu_i)}\right)}{\mu_i}\left[\gamma_i \sum_j F_{ji} + I_i - \sum_j F_{ij} - E_i\right]$	*Integration by Runge-Kutta*	
