# Supplementary material for: Non-Deterministic Modelling of Food-Web Dynamics
Source: PLoS One. 2014 Oct 9;9(10):e108243. doi: 10.1371/journal.pone.0108243 (PMC4191973; doi:10.1371/journal.pone.0108243)
Supplement: Appendix S2 — Integration of the model master equation in continuous form (eq. 1) into its discrete form, for time step t to time step t+1 (eq. 2). (PDF) [file pone.0108243.s002.pdf]

Appendix 2 – The master equation in a discrete form

We introduce time subscripts for all terms in the *master equation*, and for the sake of computational clarity, we let

$$A_{ij,t} = \gamma_i \sum_j F_{ji,t} + I_{i,t} - \sum_j F_{ij,t} - E_{i,t}. \quad (1)$$

Substituting $A_{ij,t}$ into the *master equation* gives

$$\frac{dB_{i,t}}{dt} = \gamma_i \sum_j F_{ji,t} + I_{i,t} - \sum_j F_{ij,t} - E_{i,t} - \mu_i B_{i,t}, \quad (2)$$

$$= A_{ij,t} - \mu_i B_{i,t}. \quad (3)$$

Note that (3) is a first order Ordinary Differential Equation (ODE), which is synonymous to

$$d(Be^{\mu_i t}) = A_{ij,t} e^{\mu_i t} dt. \quad (4)$$

Integrating (4) in the biomass interval $[B_{i,t} \;\; B_{i,t+1}]$ and time interval $[t \;\; t+1]$, yields

$$B_{i,t+1} e^{\mu_i (t+1)} - B_{i,t} e^{\mu t} = \int_t^{t+1} A_{ij,t'} e^{\mu_i t'} dt'. \quad (5)$$

Dividing (5) through by $e^{\mu_i (t+1)}$, and using integration by parts on the right-hand-side yields

$$B_{i,t+1} = e^{-\mu_i} B_{i,t} + \frac{(1 - e^{-\mu_i})}{\mu_i} A_{ij,t} - e^{-\mu_i (t+1)} \int_t^{t+1} \frac{e^{\mu_i t'}}{\mu_i} \left(\frac{dA_{ij,t'}}{dt'} \right) dt'. \quad (6)$$

If we assume that $A_{ij,t}$ is piecewise constant in the interval $t' \in [t \;\; t+1]$, then $\left(\frac{dA_{ij,t'}}{dt'}\right) = 0$, and (6) reduces to

$$B_{i,t+1} = e^{-\mu_i} B_{i,t} + \frac{(1 - e^{-\mu_i})}{\mu_i} A_{ij,t},$$

$$= e^{-\mu_i} B_{i,t} + \frac{(1 - e^{-\mu_i})}{\mu_i} \left[\gamma_i \sum_j F_{ji,t} + I_{i,t} - \sum_j F_{ij,t} - E_{i,t} \right]. \quad (7)$$
