# Supplementary material for: Non-Deterministic Modelling of Food-Web Dynamics
Source: PLoS One. 2014 Oct 9;9(10):e108243. doi: 10.1371/journal.pone.0108243 (PMC4191973; doi:10.1371/journal.pone.0108243)
Supplement: Appendix S3 — Comparison of the variance in rate of biomass change in the MMM and NDND models as a result of distinct formulation of the inertia constraint ( ρ ). (PDF) [file pone.0108243.s003.pdf]

Appendix 3 – Comparing the rate of biomass change

The aim of this appendix is to compare the variance in the rate of biomass change between the NDND and MMM implementations. For simplicity, we investigate the case where the rate of biomass change can assume equally probable values over an interval (i.e. uniform distribution).

Recall that minimum and maximum rates of biomass change over a unit time for NDND satisfies

$$-\rho_n B_{i,t} \leq \frac{dB_{i,t}}{dt} \leq \rho_n B_{i,t}. \tag{1}$$

The discrete version of the constraints on biomass change in MMM is given by

$$(1-\rho_m)B_t \quad \leq \quad B_{t+1} \leq (1+\rho_m)B_t \tag{2}$$

Observe that over a unit time, equation (2) is equivalent to

$$\ln(1-\rho_m)B_{i,t} \leq \frac{dB_{i,t}}{dt} \leq \ln(\rho_m+1)B_{i,t}, \quad \rho_m < 1. \tag{3}$$

We first establish an equivalence between ρ_n and ρ_m. In our implementation

$$e^{\rho_n} = (1+\rho_m). \tag{4}$$

We then substitute (4) into (3) to arrive at:

$$\ln(2-e^{\rho_n})B_{i,t} \leq \frac{dB_{i,t}}{dt} \leq \rho_n B_{i,t}, \quad \rho_n < \ln 2. \tag{5}$$

Note that the interval $[\ln(2-e^{\rho_n})B_{i,t} \;\; \rho_n B_{i,t}]$ is asymmetrical about the $B_{i,t}$, with $[\ln(2-e^{\rho_n})B_{i,t} \;\; B_{i,t}]$ being longer than $[B_{i,t} \;\; \rho_n B_{i,t}]$.

We investigate characteristics of the two different biomass rates of change assuming that this change is equiprobable over a given interval, i.e., uniform distribution. Recall that for the uniform distribution over an interval $[x_l, x_u]$, $\mathcal{U}(x_l, x_u): \;\; -\infty < x_l < x_u < \infty$, and $\forall x \in [x_l \; x_u]$,

$$\mathbf{E}(x) \;\; = \;\; 0.5(x_l + x_u), \tag{6}$$

$$\mathbf{V}(x) \;\; = \;\; \frac{(x_u - x_l)^2}{12}. \tag{7}$$

The expectation, $\mathbf{E}(\frac{dB_{i,t}}{dt})$, and variance, $\mathbf{V}(\frac{dB_{i,t}}{dt})$, of the biomass rate of change over unit time, assuming uniform distribution is sumarized in Table 1.

Table 1: Expectation and variance estimates assuming uniform distribution

Model	$\mathbf{E}(\frac{dB_{i,t}}{dt})$	$\mathbf{V}(\frac{dB_{i,t}}{dt})$	Constraints
MMM	$\frac{(\ln(2-e^{\rho_n})+\rho_n)B_{i,t}}{2} < 0$	$\frac{(\rho_n-\ln(2-e^{\rho_n}))^2 B_{i,t}^2}{12}$	$\rho_n < \ln 2$
NDND	0	$\frac{4\rho_n^2 B_{i,t}^2}{12}$	

We deduce the following:

$$\mathbf{E}(\frac{dB_{i,t}}{dt}) \in [\ln(2-e^{\rho_n})B_{i,t} \;\; B_{i,t})\,,$$

and

$$\mathbf{V}_{mmm}(\frac{dB_{i,t}}{dt}) > \mathbf{V}_{ndnd}(\frac{dB_{i,t}}{dt}).$$

Hence assuming a uniform distribution over $\frac{dB_{i,t}}{dt}$, there will be a difference in the dynamics generated by the two implementation. Wherea NDND generates a symmetrical distribution about $B_{i,t}$, MMM will generate a dynamic with biased rates of biomass change (towards the lower end of the sampling interval). MMM realizations of $\frac{dB_{i,t}}{dt}$, will also have higher variance than NDND. The higher variance is due to the fact that the difference between the lowest to highest possible realizations is much higher in MMM than in NDND.
